# Variation in inpatient investigations and treatment practices for COPD exacerbations between respiratory and internal medicine specialties: a retrospective cohort study

**DOI:** 10.3389/fmed.2026.1720006

**Published:** 2026-04-02

**Authors:** Woon Hean Keenan Chong, Wei Jun Dan Ong, Ronnie Voon Shiong Tan, Ching Yee Tan, Noel Stanley Wey Tut Tay, Adrian Ujin Yap

**Affiliations:** 1 Department of Respiratory and Intensive Care Medicine, Ng Teng Fong General Hospital, National University Health System, Singapore, Singapore; 2 Department of Respiratory Therapy, Ng Teng Fong General Hospital, National University Health System, Singapore, Singapore; 3 Department of Respiratory Medicine, Ng Teng Fong General Hospital, National University Health System, Singapore, Singapore; 4 Clinical Research Unit and Division of Dentistry, Ng Teng Fong General Hospital, National, University Health System, Singapore, Singapore; 5 Faculty of Dentistry, National University of Singapore, Singapore, Singapore; 6 Duke-14 NUS Medical School, Singapore, Singapore

**Keywords:** chronic obstructive pulmonary disease, COPD, internal medicine, internists, respiratory medicine, respiratory specialty

## Abstract

**Introduction:**

Chronic obstructive pulmonary disease (COPD) exacerbations are a major cause of hospitalization and healthcare utilization. The impact of inpatient specialty allocation on management practices remains unclear. This study examined variation in investigations and treatment practices among COPD patients admitted under respiratory medicine (RM) compared with internal medicine (IM), and evaluated clinical characteristics associated with RM admission.

**Methods:**

We conducted a retrospective cohort study of adults aged ≥40 years admitted for COPD exacerbations between January 2017 and March 2025. Patients were identified using ICD-10 COPD codes, excluding those admitted directly to ICUs or with asthma or bronchiectasis. Demographics, comorbidities, investigations, treatments, and hospitalization outcomes were analyzed. The primary outcome was variation in investigations and treatment practices between RM and IM admissions. The secondary outcome was identification of clinical characteristics associated with RM admission using multivariable logistic regression.

**Results:**

Among 6,277 COPD admissions, 3,211 (51.2%) were under RM and 3,066 (48.8%) under IM. Patients admitted under RM were younger (73.2 ± 10.7 vs. 75.6 ± 10.8 years, *p* < 0.001) and had fewer comorbidities, including ischemic heart disease (IHD), diabetes (DM), stroke, and chronic kidney disease (CKD). RM admissions underwent more respiratory-focused investigations, including chest radiography (54.2% vs. 43.5%), chest CT (8.4% vs. 5.6%), and ABG testing (23.1% vs. 8.2%). Non-invasive ventilation (17.7% vs. 4.2%) and invasive mechanical ventilation (18.2% vs. 4.3%) were more frequently used in RM admissions. Use of intravenous antibiotics, routine laboratory testing, allied health involvement, and palliative care services were more frequent in IM admissions. Descriptive differences in hospital outcomes were also observed, with RM admissions demonstrating lower in-hospital mortality (3.9% vs. 6.5%, *p* < 0.001) and shorter hospitalization duration (5.85 vs. 6.93 days, *p* < 0.001), while 30-day readmissions were similar. In multivariable analysis, older age (OR 0.97, 95% CI 0.96–0.99), male sex (OR 0.49), IHD (OR 1.60), stroke (OR 1.70), and CKD (OR 1.94 were associated with RM admission).

**Conclusion:**

Variation in investigations and treatment practices was observed between RM and IM admissions for COPD exacerbations. These differences likely reflect variation in patient characteristics, illness severity, and clinical workflows rather than differences in quality of care attributable to specialty alone.

## 1 Introduction

Chronic obstructive pulmonary disease (COPD) remains a major contributor to global morbidity and healthcare utilization, largely driven by recurrent acute exacerbations that frequently require emergency department visits and hospital admissions (1). In Singapore, COPD affects approximately 3.5% of adults aged 40 years and older and represents a substantial contributor to healthcare resource utilization, with annual readmission rates exceeding 20% despite advances in medical care (2, 3). Between 2021 and 2023, COPD ranked as the tenth leading cause of death nationally (4). Hospitalized COPD patients often present with multiple comorbidities, advanced age, frailty, and limited caregiver support, further complicating inpatient management (5). Longitudinal data suggest that approximately 75% of COPD patients experience at least one hospitalization or emergency department visit over a three-year period, and up to 45% experience two or more such episodes annually (5, 6). Although evidence-based pharmacological and supportive therapies for COPD exacerbations have evolved, the optimal inpatient care model remains uncertain. In particular, the specialty responsible for inpatient management may influence diagnostic evaluation, adherence to guideline-recommended interventions, and coordination of multidisciplinary care. These factors may ultimately affect healthcare resource utilization and patient outcomes, making the allocation of inpatient specialty services an important healthcare policy consideration.

International clinical guidelines, including those from the Global Initiative for Chronic Obstructive Lung Disease (GOLD 2025) and the UK National Institute for Health and Care Excellence (NICE 2019), outline recommended management strategies for COPD exacerbations but do not address which inpatient specialty should assume primary responsibility for hospitalized patients (7, 8). Evidence from other chronic diseases suggests that specialty-led management may improve quality of care, patient safety, and resource utilization, potentially leading to more cost-effective healthcare delivery (9, 10). However, evidence evaluating specialty-based inpatient management for COPD within Asian healthcare systems remains limited.

Although COPD exacerbations are frequently managed across different inpatient specialties, little is known about how clinical investigation patterns, treatment practices, and multidisciplinary care processes differ between respiratory medicine (RM) and internal medicine (IM) services. Therefore, we conducted a single-center retrospective study to characterize the variation in inpatient investigation patterns, treatment practices, and care processes among patients hospitalized with COPD exacerbations under RM versus IM specialties in Singapore. Additionally, we evaluated clinical characteristics associated with RM admission over IM. Our study aimed to describe unadjusted real-world differences between specialties, and any causal inference was beyond the scope of this analysis. Unlike prior analyses of this cohort that evaluated specialty-related differences in clinical outcomes using propensity-weighted methods, the present study focuses on descriptive differences in investigation patterns, treatment practices, and inpatient care processes between specialties (11).

## 2 Materials and methods

### 2.1 Study design and setting

This retrospective observational cohort study was conducted at Ng Teng Fong General Hospital (NTFGH), a 700-bed tertiary center in Singapore.

### 2.2 Patient population

From January 1, 2017 to March 31, 2025, consecutive ED admissions for acute COPD exacerbations were identified via the EPIC electronic medical record (EMR). Eligible patients were ≥40 years and coded with the International Classification of Diseases, 10th Revision (ICD-10) of COPD (J44–J42). The age threshold of 40 years was selected on the basis of established epidemiological data from multiple observational studies conducted in COPD populations (2, 12). Patients who were critically ill and admitted directly to the intensive care unit (ICU) were excluded. Patients with coexisting diagnoses of asthma or bronchiectasis were also excluded to maintain a clinically homogeneous cohort of COPD exacerbations managed in general wards. The assignment of COPD patients to RM or IM was not randomized and reflected routine clinical triage practices within the hospital. Allocation was influenced by multiple operational factors including bed availability, patient acuity, and clinical complexity. Given the retrospective nature of the dataset and the absence of a stable and established patient-level identifier suitable for reliable longitudinal linkage across admissions, repeated admissions from the same patient could not be consistently identified. Consequently, analyses were performed at the admission level rather than the patient level.

### 2.3 Data extraction

The NUHS Group Medical Informatics Office independently performed data extraction using validated scripts. Extracted data were de-identified in accordance with institutional data governance policies. The study was approved by the NTFGH IRB with waiver of informed consent (JHC-RNR-2025–1,006). The present analysis uses the same institutional COPD cohort as our previously published propensity-weighted study evaluating the association between admitting specialty and inpatient outcomes (11). However, the current study focuses on descriptive differences in investigation patterns, treatment practices, and care processes between specialties rather than causal inference on clinical outcomes.

### 2.4 Variables

Collected variables were categorized into three domains (1): baseline demographics and clinical characteristics (Table 1) (2), investigations and treatment practices (Table 2), and (3) hospitalization outcomes (Table 3). Table 1 included age, gender, body mass index (BMI), full code, ethnicity (Chinese, Malay, Indian, or others), smoking status (current or former), and influenza vaccination status in the preceding year. Full code signifies that a patient desires all possible life-saving interventions, including cardiopulmonary resuscitation, intubation and mechanical ventilation (IMV), in the event of cardiac or respiratory arrest. Comorbidities recorded included ischemic heart disease (IHD), diabetes mellitus (DM), congestive heart failure (CHF), stroke, chronic kidney disease (CKD), dementia, anxiety, and depression. Admission physiological parameters included arterial blood gas (ABG) measurements (pH and PaCO₂) and oxygen saturation (SpO₂).

**Table 1:** Baseline demographics and characteristics.

	All (*N* = 6,277)	RM (*N* = 3,211)	IM (*N* = 3,066)	*p*-value
Age, mean (+/−S.D.) years	74.38 ± 10.82	73.23 ± 10.73	75.58 ± 10.79	<0.001**
Male gender, *N* (%)	5,564 (88.6%)	2,860 (89.1%)	2,704 (88.2%)	0.074
BMI, mean (+/−S.D.) kg/m^2^	21.81 ± 2.62	21.53 ± 2.59	22.11 ± 2.62	0.075
Full code, *N* (%)	5,919 (94.3%)	3,008 (93.7%)	2,911 (94.9%)	0.031*
Ethnicity				
Chinese, *N* (%)	4,042 (64.4%)	1968 (61.3%)	2074 (67.6%)	<0.001**
Malay, *N* (%)	1,040 (16.6%)	614 (19.1%)	426 (13.9%)	<0.001**
Indian, *N* (%)	726 (11.6%)	362 (11.3%)	364 (11.9%)	0.459
Others, *N* (%)	467 (7.4%)	267 (8.3%)	200 (6.6%)	0.007**
Smoking status				
Current smoker, *N* (%)	1,376 (21.9%)	646 (20.1%)	730 (23.9%)	<0.001**
Former smoker, *N* (%)	1,437 (22.9%)	739 (23.0%)	698 (22.8%)	0.818
Comorbidities				
IHD, *N* (%)	1951 (31.1%)	844 (26.3%)	1,107 (36.1%)	<0.001**
DM, *N* (%)	1,534 (24.4%)	662 (20.6%)	872 (28.4%)	<0.001**
CHF, *N* (%)	685 (10.9%)	276 (8.6%)	409 (13.3%)	<0.001**
Stroke, *N* (%)	1,042 (16.6%)	404 (12.6%)	638 (20.8%)	<0.001**
CKD, *N* (%)	955 (15.2%)	378 (11.8%)	577 (18.8%)	<0.001**
Dementia *N* (%)	140 (2.2%)	113 (3.5%)	127 (4.1%)	0.220
Anxiety and depression, *N* (%)	141 (2.2%)	113 (3.5%)	128 (4.2%)	0.200
Admission arterial blood gas				
pH, mean (+/−S.D.)	7.35 ± 0.08	7.35 ± 0.08	7.37 ± 0.09	0.007**
PaCO₂, mean (+/−S.D.)	50.62 ± 17.24	52.66 ± 17.43	41.66 ± 13.15	<0.001**
SpO_2_ on admission, mean (+/−S.D.) %	94.39 ± 3.28	92.04 ± 3.64	95.81 ± 2.69	<0.001**
Influenza vaccine in the past year, *N* (%)	755 (12.0%)	448 (14.0%)	307 (10.0%)	<0.001**

*P value < 0.05, ** *p* value < 0.01. BMI, body mass index; CHF, congestive heart failure; CKD, chronic kidney disease; DM, diabetes mellitus; IM, internal medicine; IHD, ischemic heart disease; N, numbers; PaCO₂, partial pressure of carbon dioxide; RM, respiratory medicine; S.D., standard deviation; SpO₂, oxygen saturation; %, percentage.

**Table 2:** Inpatient investigations, ventilatory support, and treatment patterns.

	All (*N* = 6,277)	RM (*N* = 3,211)	IM (*N* = 3,066)	*p*-value
Chest imaging within 24 h admission				
Chest X-ray, *N* (%)	3,075 (49.0%)	1741 (54.2%)	1,334 (43.5%)	<0.001**
CT-chest, *N* (%)	441 (7.0%)	269 (8.4%)	172 (5.6%)	<0.001**
Total blood test				
ABG, *N* (%)	1,136 (18.1%)	741 (23.1%)	250 (8.2%)	<0.001**
FBC, *N* (%)	2,738 (43.6%)	1,201 (37.4%)	1,537 (50.1%)	<0.001**
RP, *N* (%)	1,198 (19.1%)	422 (13.1%)	776 (25.3%)	<0.001**
LFT, *N* (%)	600 (9.6%)	191 (5.9%)	409 (13.3%)	<0.001**
CRP, *N* (%)	1731 (27.6%)	749 (23.3%)	982 (32.0%)	<0.001**
Procalcitonin, *N* (%)	451 (7.2%)	265 (8.3%)	186 (6.1%)	0.001**
Respiratory support				
Non-invasive mechanical ventilation, *N* (%)	696 (11.1%)	567 (17.7%)	129 (4.2%)	<0.001**
Non-Invasive ventilator duration, mean (+/− S.D.) days	1.15 ± 0.45	1.14 ± 0.46	1.16 ± 0.39	0.700
Mechanical ventilator, N (%)	715 (11.4%)	584 (18.2%)	131 (4.3%)	<0.001**
Mechanical ventilator duration, mean (+/− S.D.) days	1.42 ± 1.35	1.41 ± 1.33	1.44 ± 1.41	0.853
Systemic antibiotic				
Oral, *N* (%)	3,215 (51.2%)	2034 (63.3%)	1,181 (38.5%)	<0.001**
I.V., *N* (%)	2,283 (36.4%)	1,122 (34.9%)	1,161 (37.9%)	0.016*
Duration of antibiotic, mean (+/− S.D.) days	3.64 ± 4.34	3.46 ± 4.45	3.88 ± 4.19	0.002**
Allied health involvement				
Physiotherapy, *N* (%)	4,226 (67.3%)	2001 (62.3%)	2,225 (72.6%)	<0.001**
Occupational therapy, *N* (%)	3,512 (56.0%)	1,478 (46.0%)	2034 (66.3%)	<0.001**
Medical social worker, *N* (%)	1,633 (26.0%)	744 (23.2%)	889 (29.0%)	<0.001**
Palliative care				
Palliative care referral, *N* (%)	146 (2.3%)	62 (1.9%)	84 (2.7%)	0.033*
Opioids, *N* (%)	863 (13.7%)	378 (11.8%)	485 (15.8%)	<0.001**

*P value < 0.05, ** *P* value < 0.01. ABG, arterial blood gas; CRP, C-reactive protein; CT, computed tomography; FBC, full blood count; IM, internal medicine; I.V., intravenous; LFT, liver function test; N, numbers; paCO2, partial pressure of carbon dioxide; RP, renal panel; RM; respiratory medicine; S.D., standard deviation, %, percentage.

**Table 3:** Clinical Outcomes.

Hospitalization outcomes	All (*N* = 6,277)	RM (*N* = 3,211)	IM (*N* = 3,066)	*P*-value
All-cause in-hospital mortality, N (%)	325 (5.2%)	126 (3.9%)	199 (6.5%)	<0.001**
Duration of hospitalization, mean (+/−S.D.) days	6.38 ± 8.49	5.85 ± 8.41	6.93 ± 8.54	<0.001**
30-day readmission, N (%)	1,278 (20.4%)	676 (21.1%)	602 (19.6%)	0.165

*P value < 0.05, ** *p* value < 0.01. *p*-values reflect unadjusted between-group comparisons and are presented for descriptive purposes only. Abbreviations: IM, internal medicine; N, numbers; RM, respiratory medicine; S.D., standard deviation, %, percentage.

Table 2 included total chest X-ray and chest computed tomography (CT) performed within 24 h of admission. The chest imaging investigations were performed during the first 24 h of admission period and do not include chest imaging performed prior to ED admission or at external facilities, such as another institution or outpatient clinics. Total number of laboratory investigations performed during hospitalization were also recorded and included ABG, full blood count (FBC), renal panel (RP), liver function test (LFT), C-reactive protein (CRP), and procalcitonin. Respiratory support modalities were evaluated, including use of non-invasive ventilation (NIV) and IMV. Treatments including systemic antibiotics (oral and intravenous [I.V.]) were recorded along with duration of therapy. Allied health involvement (physiotherapy, occupational therapy, and medical social worker) and palliative care interventions (referrals and opioid use) were also documented. Table 3 included all-cause in-hospital mortality, duration of hospitalization, and 30 days readmission. The 30-day readmissions rates included COPD patients admitted to our institution as well as to other public hospitals within the regional healthcare network. Competing risks, including death, were not formally modeled, and therefore, readmission estimates should be interpreted with caution.

The primary outcome was to characterize the variation in inpatient investigation patterns, treatment practices, and care processes between RM and IM admissions. The secondary outcome was the identification of clinical characteristics associated with admission under RM compared with IM, evaluated using logistic regression analyses.

### 2.5 Statistical analysis

Continuous variables were summarized as mean ± SD and compared via Student’s t-test, while categorical variables were expressed as counts and % and compared using the Chi-square test. These unadjusted comparisons were used to evaluate observed variation in investigations and treatment practices between admissions under RM and IM. To identify clinical characteristics associated with admission under RM compared with IM, multivariable logistic regression analyses were performed. Results are reported as odds ratios (ORs) with 95% confidence intervals (CIs). For continuous variables with <5% missing data, mean imputation was performed. Variables with >10% missingness were excluded from regression analyses to minimize potential bias. All the statistical tests were conducted in a two-sided manner, and a *p* value ≤ 0.05 was considered statistically significant. Analyses were performed via SPSS Statistics version 25.0 (IBM Corp., Armonk, NY, USA).

## 3 Results

### 3.1 Baseline demographics and clinical characteristics

A total of 6,277 patients were included, with 3,211 (51.2%) admitted under RM and 3,066 (48.8%) under IM. The COPD patients admitted under RM were younger than those admitted under IM (73.2 ± 10.7 vs. 75.6 ± 10.8 years, *p* < 0.001) (Table 1). Gender distribution did not differ in both groups, with a predominance of males (89.1% in RM vs. 88.2% in IM, *p* = 0.074). BMI was similar in those admitted under RM than in IM (21.5 ± 2.6 vs. 22.1 ± 2.6 kg/m^2^, *p* = 0.075). A slightly lower proportion of patients admitted under RM had full code status compared with IM (93.7% vs. 94.9%, *p* = 0.031). The ethnic distribution differed with patients admitted under RM were more frequently Malay (19.1% vs. 13.9%, *p* < 0.001) and less often Chinese (61.3% vs. 67.6%, *p* < 0.001) than those admitted under IM, while the proportion of Indian patients was similar between groups (11.3% vs. 11.9%, *p* = 0.459). Patients categorized as other ethnicities were slightly more common among RM admissions (8.3% vs. 6.6%, *p* = 0.007).

Current smoking was more common among patients admitted under IM (23.9% vs. 20.1%, *p* < 0.001), whereas the proportion of former smokers were similar between groups (23.0% vs. 22.8%, *p* = 0.818) (Table 1). Several comorbidities were more frequently observed among patients admitted under IM, including IHD (36.1% vs. 26.3%), DM (28.4% vs. 20.6%), CHF (13.3% vs. 8.6%), stroke (20.8% vs. 12.6%), and CKD (18.8% vs. 11.8%) (all *p* < 0.001). The prevalence of dementia and anxiety or depression did not differ significantly between the groups. Admission ABG measurements showed small but statistically significant differences between groups (Table 1). Patients admitted under RM had slightly lower mean pH values (7.35 ± 0.08 vs. 7.37 ± 0.09, *p* = 0.007) and higher mean PaCO₂ levels (52.7 ± 17.4 vs. 41.7 ± 13.2 mmHg, *p* < 0.001) compared with those admitted under IM. SpO_2_ on admission was lower among RM admissions (92.0 ± 3.6% vs. 95.8 ± 2.7%, *p* < 0.001). Influenza vaccination in the preceding year was more prevalent among patients admitted under RM compared with IM (14.0% vs. 10.0%, *p* < 0.001).

### 3.2 Investigations and treatment practices

COPD patients admitted under RM underwent more respiratory-specific investigations within the first 24 h of admission. Chest radiography was more frequently performed among RM admissions compared with IM (54.2% vs. 43.5%, *p* < 0.001), as was chest CT (8.4% vs. 5.6%, *p* < 0.001) (Table 2). In contrast, several routine laboratory tests were more frequently performed among patients admitted under IM. These included FBC (50.1% vs. 37.4%), RP (25.3% vs. 13.1%), LFT (13.3% vs. 5.9%), and CRP (32.0% vs. 23.3%) (all *p* < 0.001). ABG testing was more commonly performed in the RM group (23.1% vs. 8.2%, *p* < 0.001). Procalcitonin testing was slightly more common among RM admissions compared with IM (8.3% vs. 6.1%, *p* = 0.001). Use of ventilatory support differed between the two services. NIV was more frequently used among patients admitted under RM compared with IM (17.7% vs. 4.2%, *p* < 0.001) (Table 2). Similarly, IMV was more common among RM admissions (18.2% vs. 4.3%, *p* < 0.001). However, the duration of ventilatory support did not differ significantly between the groups for either NIV (1.14 ± 0.46 vs. 1.16 ± 0.39 days, *p* = 0.700) or IMV (1.41 ± 1.33 vs. 1.44 ± 1.41 days, *p* = 0.853).

Differences were also observed in systemic antibiotic use. Oral antibiotics were more frequently prescribed among RM admissions compared with IM (63.3% vs. 38.5%, *p* < 0.001) (Table 2). In contrast, IV antibiotics were slightly more common among IM admissions (37.9% vs. 34.9%, *p* = 0.016). The mean duration of antibiotic therapy was slightly longer among IM admissions compared with RM (3.88 ± 4.19 vs. 3.46 ± 4.45 days, *p* = 0.002). Allied health involvement was more frequently observed among patients admitted under IM. Physiotherapy (72.6% vs. 62.3%), occupational therapy (66.3% vs. 46.0%), and medical social worker support (29.0% vs. 23.2%) were all more common among IM admissions (all *p* < 0.001). Palliative care involvement was also slightly more frequent among IM admissions, including palliative care referrals (2.7% vs. 1.9%, *p* = 0.033) and opioid use (15.8% vs. 11.8%, *p* < 0.001).

### 3.3 Hospitalization outcomes

Differences were observed in several hospitalization outcomes between patients admitted under RM and IM (Table 3). However, these analyses were descriptive and not adjusted for baseline differences. All-cause in-hospital mortality was lower among patients admitted under RM compared with those admitted under IM (3.9% vs. 6.5%, *p* < 0.001). Patients admitted under RM also had a shorter mean duration of hospitalization compared with IM admissions (5.85 ± 8.41 vs. 6.93 ± 8.54 days, *p* < 0.001). The proportion of patients readmitted within 30 days did not differ significantly between the two groups (21.1% in RM vs. 19.6% in IM, *p* = 0.165).

### 3.4 Logistic regression analysis of clinical characteristics associated with RM admission

Multivariable logistic regression analysis was performed to evaluate clinical characteristics associated with admission under RM compared with IM (Table 4). Older age was associated with lower odds of RM admission (OR 0.97, 95% CI 0.96–0.99, *p* < 0.001). Male sex was also associated with lower odds of admission under RM compared with IM (OR 0.49, 95% CI 0.30–0.80, *p* = 0.004). Several comorbidities were associated with higher odds of RM admission, including IHD (OR 1.60, 95% CI 1.16–2.20, *p* = 0.004), stroke (OR 1.70, 95% CI 1.15–2.52, *p* = 0.008), and CKD (OR 1.94, 95% CI 1.25–3.00, *p* = 0.003). Higher arterial pH was associated with greater odds of RM admission (OR 1.30, 95% CI 1.21–1.39, *p* < 0.001). In contrast, PaCO₂ levels and SpO₂ on admission were not significantly associated with admission specialty. Other variables, including BMI, DM, CHF, ethnicity, smoking status, influenza vaccination in the preceding year, and full code status, were not significantly associated with RM admission in the adjusted model.

**Table 4:** Logistic regression analysis for clinical characteristics associated with RM admission.

Variable	Adjusted OR	95% CI	P-value
Age	0.97	0.96–0.99	<0.001**
Male gender	0.49	0.30–0.80	0.004*
BMI	0.99	0.97–1.02	0.573
Full code status	0.90	0.46–1.75	0.759
Ethnicity			
Chinese ethnicity	1.42	0.88–2.29	0.151
Malay ethnicity	1.09	0.63–1.89	0.763
Indian ethnicity	1.44	0.81–2.58	0.214
Smoking status			
Current smoker	1.29	0.87–1.90	0.203
Former smoker	1.11	0.76–1.61	0.590
Comorbidities			
IHD	1.60	1.16–2.20	0.004*
DM	1.34	0.93–1.92	0.114
CHF	1.35	0.82–2.24	0.243
Stroke	1.70	1.15–2.52	0.008*
CKD	1.94	1.25–3.00	0.003*
Admission arterial blood gas			
Arterial pH	1.30	1.21–1.39	<0.001**
PaCO₂	1.00	0.999–1.003	0.885
SpO₂ on admission	0.99	0.98–1.01	0.468
Influenza vaccination in the past year	1.16	0.73–1.83	0.534

*P value < 0.05, ** *P* value < 0.01. BMI, body mass index; CHF, congestive heart failure; CI, confidence interval; CKD, chronic kidney disease; DM, diabetes mellitus; IM, internal medicine; IHD, ischemic heart disease; N, numbers; PaCO₂, partial pressure of carbon dioxide; RM, respiratory medicine; S.D., standard deviation; SpO₂, oxygen saturation; %, percentage.

## 4 Discussion

### 4.1 Principal findings

This study provides one of the largest evaluations in an Asian healthcare setting examining differences in inpatient diagnostic and treatment practices for COPD exacerbations across RM and IM services. Across 6,277 admissions over an eight-year period (RM 51.2%, IM 48.8%), meaningful differences were observed in patient characteristics, investigations, and treatment practices between specialties. Patients admitted under RM were generally younger and had fewer comorbidities, whereas those admitted under IM more frequently had IHD, DM, CHF, stroke, and CKD. IM admissions also included a higher proportion of patients of Chinese ethnicity and current smokers. Multivariable logistic regression analysis further identified factors associated with RM admission. Older age and male sex were associated with lower odds of RM admission, whereas IHD, stroke, and CKD were associated with higher odds of admission under RM. BMI, ethnicity, smoking status, influenza vaccination, and admission SpO₂ were not significantly associated in the adjusted model. These findings suggest that specialty allocation may partly reflect differences in clinical presentation and comorbidity profiles among hospitalized COPD patients.

Differences in practice patterns were also observed between specialties. RM care was characterized by greater use of respiratory-focused investigations, including chest imaging, ABG analysis, and procalcitonin testing, as well as higher use of oral antibiotics and more frequent initiation of ventilatory support, including NIV and IMV. However, IM care more often involved routine laboratory testing, IV antibiotics with longer treatment durations, and more frequent involvement of allied health and palliative care services. Despite the higher utilization of ventilatory support among RM admissions, patients admitted under RM demonstrated lower all-cause in-hospital mortality and shorter duration of hospitalization. In summary, these findings highlight variation in inpatient COPD care processes across specialties and underscore potential opportunities for harmonizing care pathways rather than demonstrating causal differences in clinical effectiveness.

### 4.2 Context within global evidence

International evidence evaluating the influence of admitting specialty on COPD inpatient management remains limited. Small prospective studies from Thailand and Israel have reported shorter duration of hospitalization under RM-led care, although effects on mortality, readmissions, and healthcare utilization were inconsistent (13, 14). Earlier audit data from the United Kingdom similarly demonstrated that COPD patients admitted under RM were more likely to receive guideline-concordant discharge planning, smoking cessation counseling, and referrals for follow-up and pulmonary rehabilitation (15). Our findings extend these observations to a large Asian cohort. Healthcare delivery models, specialist availability, and sociocultural factors differ substantially between Asian and Western healthcare systems. The ethnic distribution in our cohort, predominantly Chinese (65%), followed by Malay (17%) and Indian (12%) patients, largely reflects Singapore’s population demographics (16). However, differences in ethnic representation across specialties may also reflect broader socioeconomic factors and healthcare-seeking behaviors. Prior studies in Singapore have shown that Chinese populations generally have higher educational attainment and health literacy, which may facilitate earlier healthcare engagement, while minority groups may experience greater socioeconomic barriers, lower health literacy, or delayed healthcare-seeking behavior (17). Although the present study was not designed to examine these sociocultural determinants, they may partly contribute to the observed variation in specialty allocation and warrant further investigation.

### 4.3 Differences in care models, treatment patterns, and clinical workflows

The two specialties appear to reflect complementary models of inpatient COPD care. RM specialty typically emphasizes respiratory pathophysiology, early diagnostics, and timely escalation to ventilatory support, involving NIV and IMV (9). However, IM specialty commonly manages older patients with greater multimorbidity and frailty, focusing on broader medical stabilization, rehabilitation, and palliative care planning (10). The observed patterns in our cohorts, involving more ABGs, chest imaging, and ventilatory support under RM, but greater use of IV antibiotics, allied health services, and palliative care under IM are consistent with these different clinical focuses. Similar patterns have been reported in an European audit study, where respiratory specialist care was associated with higher utilization of ABG testing and NIV (18). However, specialist-led care may also increase healthcare costs through greater use of advanced diagnostics and respiratory-specific interventions (19).

From a health-system perspective, these differences highlight opportunities for integrated care models rather than strict specialty-based pathways (20). Potential strategies include early respiratory consultations for high-risk COPD admissions, multidisciplinary RM–IM ward rounds, and standardized inpatient COPD care bundles (21). Such bundles may include inhaler technique assessment, smoking cessation support, vaccination review, medication reconciliation, pulmonary rehabilitation referral, and structured outpatient follow-up (22–24). Integration of respiratory-trained nurses, physiotherapists, and pharmacists into general wards may further support COPD management (25, 26). Lastly, EMR-based prompts and standardized order sets could facilitate consistent application of COPD care bundles across specialties (26). These system-level approaches may help harmonize care delivery while maintaining cost-effectiveness.

### 4.4 Interpretation of hospital outcomes

Although patients admitted under RM required ventilatory support more frequently, they also demonstrated lower in-hospital mortality and shorter duration of hospitalization compared with those admitted under IM. These outcome differences should be interpreted cautiously because the present analysis was not designed to evaluate causal relationships between admitting specialty and clinical outcomes. Instead, these findings likely reflect differences in baseline patient characteristics, comorbidity burden, illness severity, and institutional triage practices between specialties. First, baseline differences in patient characteristics likely contributed to the difference in outcomes. Patients admitted under RM were younger and had fewer systemic comorbidities, such as IHD, DM, stroke, and CKD, which are known to influence hospitalization outcomes in COPD exacerbations (27). Second, lower SpO₂ and higher PaCO₂ on admission may reflect a respiratory-predominant exacerbation phenotype rather than multisystem illness (28). However, patients admitted under IM more frequently had multiple cardiometabolic comorbidities that may complicate inpatient management and prolong recovery (10, 27). Third, differences in clinical workflows may influence outcomes, including earlier recognition of respiratory failure with more rapid initiation of ventilatory support within RM services (10). Additionally, local triage practices may preferentially allocate patients with primary respiratory failure to RM services, whereas patients with complex multisystem comorbidities may be managed under IM. Given the observational design of this study and the absence of key disease-severity measures such as spirometry, GOLD staging, BODE index components, and long-term oxygen use, these findings should not be interpreted as evidence that specialty admission alone improves outcomes (15, 29).

The mortality rates observed (RM 3.9%, IM 6.5%) in our cohort align with pooled registry estimates (6.2%) (30) and are lower than ICU cohorts (11.5%) (31), which is expected given our exclusion of patients admitted directly from ED to ICU. The interpretation of outcome differences should also consider the potential impact of survivorship bias, which arises when patients requiring immediate ICU admission are excluded from the analysis and patient who died early had shorter duration of hospitalization. The exclusion of ICU admissions was taken to maintain a more homogeneous ward-based cohort and avoid conflating ward-level management with critical care pathways. However, these patients likely represent the most severely ill subgroup, and their exclusion from the analysis may lead to a biased study population. Consequently, observed outcomes may underestimate overall disease severity. Additionally, if critically ill patients were preferentially triaged to one specialty, this could further influence between-group comparisons.

The 30-day readmission rate in this study encompassed readmissions to our institution as well as to other public hospitals within the regional healthcare network. Singapore’s public healthcare system operates within an integrated regional network in which hospitals share a unified EMR platform termed EPIC. This system enables the capture of inpatient admissions, including readmissions, across institutions within the same healthcare cluster (32, 33). As such, readmissions occurring at other public hospitals within the regional network were captured and included in our analysis. However, readmissions to private healthcare institutions not utilizing EPIC EMR are not captured and may therefore lead to a modest underestimation of overall readmission rates. Given that the majority of inpatient care in Singapore is delivered within the public healthcare system, the impact of this limitation is likely small (34). Due to the retrospective nature of the dataset and the absence of a stable patient-level identifier, analyses were conducted at the admission level rather than the individual patient level. As a result, repeated readmissions from the same patient reflect repeated episodes of healthcare utilization rather than independent events. Standard statistical approaches that do not account for within-patient correlation may underestimate variance and overstate precision. Future studies incorporating unique patient identifiers and longitudinal analytic methods would allow for modeling of repeated admissions and provide more accurate estimates of readmission risk.

### 4.5 Strengths and limitations

This study has several strengths. It includes a large cohort of more than 6,000 COPD admissions over an eight-year period and leverages a comprehensive EMR dataset. The stringent inclusion criteria excluding patients with asthma/bronchiectasis and those admitted directly to ICU reduced clinical heterogeneity, focusing analysis on ward-level COPD care. The use of an integrated healthcare network enabled capture of readmissions across public hospitals within the same regional cluster, enhancing outcome completeness.

Nevertheless, several limitations should be acknowledged. First, confounding by indication is inherent to this retrospective design. Patients admitted under RM were generally younger and less comorbid than those admitted under IM, and these differences may partly explain observed outcomes. Second, admission specialty was not randomized and reflected routine clinical triage influenced by illness severity, comorbid burden, and operational factors. Patients admitted directly to the ICU were excluded to maintain a ward-based cohort, which may have introduced selection bias by excluding the most severely ill patients. As a result, observed outcomes may underestimate overall disease severity and should be interpreted cautiously. Third, analyses were conducted at the admission level rather than the individual patient level because unique patient identifiers suitable for longitudinal linkage were unavailable. Consequently, repeated admissions from the same patient could not be accounted for, and may recurrent events rather than independent observations. Fourth, the extended study period (2017–2025) encompasses evolving healthcare practices and the impact of the COVID-19 pandemic, which may have influenced admission patterns and outcomes. Fifth, key measures of COPD severity, including long-term oxygen use, Charlson comorbidity index, BODE index, GOLD stage, spirometry and other pulmonary function tests (PFTs) parameters were unavailable due to limitations in data integration across systems. This limitation reflects the fragmented storage of spirometry and PFT results across different institutional systems rather than an omission in study design. Their absence restricts risk adjustment and may partially explain observed associations. Finally, readmission outcomes were analyzed descriptively, ignoring competing risks like death. Observed differences in 30-day readmission rates may reflect survivorship rather than true post-discharge risk.

## 5 Conclusion

In this large retrospective cohort of COPD exacerbation admissions, meaningful differences were observed in patient characteristics, inpatient investigations, and treatment practices between RM and IM services. RM admissions were characterized by greater use of respiratory-focused investigations and ventilatory support, whereas IM admissions more frequently involved routine laboratory testing, IV antibiotics, and allied health and palliative care involvement. Patients admitted under RM also demonstrated lower in-hospital mortality and shorter duration of hospitalization. However, these differences should be interpreted in the context of underlying variations in patient case mix, comorbidity burden, and clinical workflows rather than evidence of superior care attributable to specialty alone. Future studies employing prospective designs, standardized triage criteria, and causal inference methods may better clarify the independent impact of admitting specialty on inpatient management and outcomes among patients hospitalized for COPD exacerbations.

## Data Availability

The original contributions presented in the study are included in the article/supplementary material, further inquiries can be directed to the corresponding author.
